# Advanced Age as a Predictor of Survival and Weaning in Venoarterial Extracorporeal Oxygenation: A Retrospective Observational Study

**DOI:** 10.1155/2017/3505784

**Published:** 2017-04-06

**Authors:** WooSurng Lee, YoHan Kim, HyunHee Choi, HyoungSoo Kim, SunHee Lee, HeeSung Lee, HyunKeun Chee, JunSeok Kim, JaeJoon Hwang, SongAm Lee, YongHun Kim, SeongJoon Cho, SeMin Ryu, SungMin Park

**Affiliations:** ^1^Department of Thoracic and Cardiovascular Surgery, School of Medicine, Konkuk University Chungju Hospital, Konkuk University, Chungju-si, Chungbuk, Republic of Korea; ^2^Department of Internal Medicine and Division of Cardiology, College of Medicine, Hallym University Chuncheon Sacred Heart Hospital, Hallym University, Chuncheon-si, Gangwon-do, Republic of Korea; ^3^Department of Thoracic and Cardiovascular Surgery, College of Medicine, Hallym University Sacred Heart Hospital, Hallym University, Gyeonggi-do, Republic of Korea; ^4^Department of Thoracic and Cardiovascular Surgery, College of Medicine, Hallym University Dongtan Sacred Heart Hospital, Hallym University, Gyeonggi-do, Republic of Korea; ^5^Department of Thoracic and Cardiovascular Surgery, School of Medicine, Konkuk University Seoul Hospital, Konkuk University, Seoul, Republic of Korea; ^6^Department of Surgery, School of Medicine, Konkuk University Chungju Hospital, Konkuk University, Chungju-si, Chungbuk, Republic of Korea; ^7^Department of Thoracic and Cardiovascular Surgery, School of Medicine, Kangwon National University, Chuncheon-si, Gangwon-do, Republic of Korea

## Abstract

*Background*. In most reports on ECMO treatment, advanced age is classified as a contraindication to VA ECMO. We attempted to investigate whether advanced age would be a main risk factor deciding VA ECMO application and performing VA ECMO support. We determined whether advanced age should be regarded as an absolute or relative contraindication to VA ECMO and could affect weaning and survival rates of VA ECMO patients.* Methods.* VA ECMO was performed on 135 adult patients with primary cardiogenic shock between January 2010 and December 2014. Successful weaning was defined as weaning from ECMO followed by survival for more than 48 hours.* Results*. Among the 135 patients, 35 survived and were discharged uneventfully, and the remaining 100 did not survive. There were significant differences in survival between age groups, and older age showed a lower survival rate with statistical significance (*P* = .01). By multivariate logistic regression analysis, age was not significantly associated with in-hospital mortality (*P* = .83) and was not significantly associated with VA ECMO weaning (*P* = .11).* Conclusions.* Advanced age is an undeniable risk factor for VA ECMO; however, patients of advanced age should not be excluded from the chance of recovery after VA ECMO treatment.

## 1. Introduction

Extracorporeal life support (ECLS) is a general terminology used to describe support of cardiac or pulmonary function with a mechanical device. Initial experience with extracorporeal membrane oxygenation (ECMO) was employed for acute respiratory distress syndrome, with the early experience with ECMO predominantly reported in neonatal and pediatric cohorts [1–3]. ECMO has remarkably progressed over the past several decades and has been accepted as an invaluable tool to treat children and adults with severe cardiac and/or pulmonary dysfunction refractory to conventional management [4–12]. This outstanding achievement and an immense increase in the number of patients who were treated with ECMO, as well as expansion of ECMO indications, raise ethical issues and dilemmas on which patients should be treated with ECMO and when ECMO support should be stopped [13]. The ELSO described that advanced age is an absolute or relative contraindication to ECMO in adult cardiac failure cases and that although advanced age is no specific contraindication to ECMO, the risk of mortality increases with age in adult respiratory failure cases [14, 15]. Physicians face a therapeutic dilemma as to whether ECMO support should be initiated to treat patients of advanced age with severe cardiac and pulmonary dysfunction refractory to conventional management. Because ECMO supplies supportive therapy rather than disease-modifying treatment, the best treatment outcome could be obtained when appropriate patients, relevant ECMO types, and proper configurations are chosen [16, 17]. Established ELSO indications in adults show that advanced age belongs to an absolute or relative contraindication to venoarterial (VA) ECMO. In most reports on ECMO treatment, advanced age is classified as an absolute contraindication to VA ECMO, so that VA ECMO is not recommended for patients of advanced age. Unfortunately, since there are no definite age criteria for VA ECMO, it is essential to make a decision as to whether age is actually a primary risk of VA ECMO and what would be the optimal age for VA ECMO. We attempted to investigate whether advanced age would be a main risk factor for deciding VA ECMO application and performing VA ECMO support. Additionally, we attempted to determine whether advanced age should be regarded as an absolute or relative contraindication to VA ECMO and could affect weaning and survival rates of VA ECMO patients.

## 2. Subjects and Methods

### 2.1. Study Patients

The ECMO support program was first initiated at a single medical center in January 2006, and VA ECMO was performed on 135 adult patients with primary cardiogenic shock between January 2010 and December 2014. All the patients were aged ≥18 years. They received VA ECMO at a single medical center by a single ECMO team directed mainly by cardiothoracic surgeons, which was performed for refractory cardiogenic shock and various medical conditions (Table 1). To avoid selection bias, this study excluded patients with respiratory failure undergoing VV ECMO. ELSO indications for VA ECMO in adults were applied; however, advanced age was not considered an absolute or relative contraindication. Successful weaning was defined as weaning from ECMO followed by survival for more than 48 hours. Survival was defined as weaning from ECMO and improvement in an underlying clinical condition followed by discharge from the hospital. This study was approved by the Institutional Review Board (2013-105), and informed consent was waived due to its retrospective study design.

### 2.2. Data Collection

We retrospectively analyzed all patients who underwent VA ECMO support. They were registered in a unique ECMO register form, and additional data were obtained from the medical records of 135 patients. Pre-ECMO characteristics, including age, sex, body mass index (BMI), medical history, and underlying disease, as well as pre-ECMO information about cardiac arrest, including the location of arrest, extracorporeal cardiopulmonary resuscitation (ECPR) time, and complications associated with cardiopulmonary resuscitation (CPR) or ECMO CPR, were obtained. Additionally, pre-ECMO data—including laboratory findings, sepsis-related organ failure assessment (SOFA) score, pre-ECMO simplified acute physiology score II (SAPS II), door-to-ECMO time, and ECMO duration—and post-ECMO data—ECMO mode, anticoagulation, duration, continuous renal replacement therapy, transfusion, and length of hospital stay—were retrospectively assessed. In cases of cardiac arrest, heart rate and Glasgow Coma Scale score were calculated at the lowest value. If a patient died within 24 hours, the variable urine output was estimated by multiplying average hourly urine output (total urine output divided by total time) by 24 [18, 19]. Acute kidney injury was defined according to the acute kidney injury network (AKIN) and the risk, injury, failure, loss of kidney function, and end-stage kidney disease (RIFLE) classification system which comprises individual serum creatinine (Cr) levels and urine output [20, 21].

### 2.3. Indications

The application of ECMO support is usually considered in critically ill patients and indicated when the mortality exceeds 80% without ECMO support [22]. The ELSO recommended that indications for ECLS must be limited to severe acute cardiac or respiratory failure with high mortality risk despite optimal conventional therapy. The application of ECLS is first considered at 50% mortality risk, and then it is indicated in most clinical conditions at 80% mortality risk. Disease severity and mortality risk were measured as precisely as possible using measurements for appropriate age and organ failure [23]. Main indications for VA ECMO were as follows: (1) refractory cardiogenic shock with a systolic blood pressure of <80 mm Hg despite appropriate conventional treatment, or cardiogenic shock combined with septic or neurogenic shock; (2) cardiac arrest that did not respond to returned spontaneous circulation within 10 minutes of CPR; and (3) recurrent cardiac arrest within 20 minutes after return of spontaneous circulation in spite of optimal CPR. One of the most common contraindications to VA ECMO is cerebral hemorrhage, because it requires anticoagulation and may aggravate cerebral hemorrhage. Another contraindication to VA ECMO is severe immunosuppression due to systemic inflammation, unwitnessed cardiac arrest, terminal-stage conditions, including terminal malignancy, or low possibility of full recovery [22]. VA ECMO is most frequently adopted in cardiogenic shock caused by a variety of etiologies, such as postmyocardial infarction, fulminant myocarditis, peripartum cardiomyopathy, cardiac depression aggravated by septic shock, decompensated heart failure, and most commonly occurring postcardiotomy shock, failure to wean off CPB, and extracorporeal CPR [24].

### 2.4. ECMO and Cannulation

Each ECMO support was performed by a team of simultaneous cardiothoracic surgeons and intensivist physicians, and absolute hemodynamic criteria were applied in ECMO initiation. Before cannulation, patients received 3,000 to 5,000 IU of intravenous unfractionated heparin. After confirming activated clotting time greater than 180 seconds, peripheral cannulation for ECMO was performed. ECMO was instituted via peripheral cannulation, in which the femoral artery and vein were cannulated with single-lumen cannulae, for drainage from the infradiaphragmatic inferior vena cava and return into the iliac artery. Transthoracic central or carotid artery cannulation was not performed. Peripheral cannulation was done in all patients, and all these procedures were performed by both simultaneous cardiothoracic surgeons and intensivists [25]. A venous cannula was placed just below the right atrium through the femoral vein, and an arterial cannula was placed in the iliac artery via the femoral artery using the Seldinger technique under the guidance of ultrasound [26]. Our principle for cannulation is the application of ultrasonography and fluoroscopy. Prior to cannulation, ultrasonography was performed to assess vessel size and patency at potential sites of cannulation. For venous cannulation, the right internal jugular and both femoral veins were imaged in the supine position, and the compressibility and Doppler color flow were used to verify vein diameter and patency. For femoral arterial cannulation, the arterial diameter was also determined using the same maneuver used to measure the vein diameter with ultrasonography. Cannulae were selected to be at least of 2-Fr size smaller than the calculated vessel size in order to maintain distal limb perfusion of ipsilateral femoral arterial cannulation. Retrograde cannulation of the common or superficial femoral artery was not routinely performed. Only patients with femoral artery stenosis and insufficient diameter underwent the percutaneous approach, in which a 7- or 8-Fr retrograde cannula was inserted into the femoral artery and positioned distally to prevent the development of distal limb ischemia [27]. Patients without retrograde cannulation in the common or superficial femoral artery were more carefully identified and monitored for the development of limb ischemia by clinical examination or pulse oximetry as well as confirmative ultrasonography. Imaging was withheld during rapid deployment ECMO, such as the patients on extracorporeal CPR or the patients in whom delay in ECMO initiation could lead to significant deterioration or impending cardiac arrest.

### 2.5. ECMO Circuit System

All patients on VA ECMO support were managed on a Bioline heparin-coated Quadrox PLS circuit system (Maquet Cardiopulmonary AG, Hirrlingen, Germany). A single-stage cannula was applied for arterial cannulation (18 cm in length; 15-, 17-, 19-, and 21-Fr) manufactured by Medtronic Bio-Medicus (Medtronic, Inc., Minneapolis, MN, USA), and a multistage cannula was applied for venous cannulation (60 cm in length; 19-, 21-, 23-, and 25-Fr) manufactured by Medtronic Bio-Medicus. The venous drainage and arterial return cannulae were inserted by the percutaneous Seldinger technique. In VA ECMO, the tip of the venous drainage cannula was positioned in the right atrium, and the arterial reinfusion cannula was inserted into the femoral artery. ECMO blood flow was adjusted to meet the hemodynamic and oxygen requirements of the patient. In some patients with renal insufficiency, continuous renal replacement therapy was integrated into the circuit.

### 2.6. ECMO Maintenance

The ultimate goal of ECMO application is to preserve all organs and to gain times for recovery. The metabolic panel and all laboratory test results were verified daily to identify proper organ perfusion and oxygenation. Arterial blood gas analysis (ABGA) and coagulation panels were obtained hourly, especially during the first day of ECMO support, and were verified at 2- or 4-hour intervals from the second day of ECMO support. The protective lung ventilation mode was preferred to allow pulmonary recovery if possible and necessary. According to hemodynamics and ABGA results, ECMO blood flow was adequately adjusted to maintain a cardiac index of 2.4 L/min/m^2^, a mixed venous oxygen saturation (SvO_2_) level of around 70%, and a mean arterial blood pressure of 70–75 mm Hg, and the oxygen flow (FiO_2_) was controlled to maintain a postoxygenator partial oxygen pressure of ≥200 mm Hg. Inotropes and vasoconstrictors were administered to maintain baseline vital signs and to facilitate optimal myocardial recovery. Intra-aortic balloon pump (IABP) support was employed in cardiogenic shock patients to decrease afterload, increase coronary perfusion, and increase pulsatility. On the one hand, ECMO blood flow was adjusted sufficiently to keep enough systemic perfusion measured by urine output, plasma lactic acid levels, and mixed venous saturation. On the other hand, ECMO blood flow was adjusted to maintain enough lung circulation. Patients who received extracorporeal CPR and who exhibited a Glasgow Coma Scale score of <9 (eye response: eye opening to speech, motor: obedience to commands, verbal: intubated state) were applied to hypothermic therapy by maintaining their body temperature at 33°C-34°C for 24 hours and without administration of sedative drugs [27, 28]. If the patient in hypothermia showed a Glasgow Coma Scale of ≥9, their body temperature was increased at 0.2°C/hour, and the patient was immediately given sedative drugs to preserve neurologic function. Hematocrit was maintained at a level of 30%–35%. Platelet count was maintained at >100,000/*μ*L, and platelets were administered to maintain this level. Fresh frozen plasma, coagulation factors, and antithrombin III were administered on the basis of the laboratory test results and clinical necessity. To keep anticoagulation and to prevent clot formation in the oxygenator during ECMO support, intravenous heparin or nafamostat mesilate (SK Chemicals Life Science Biz., Seoul, Korea, licensed by Torii Pharmaceutical Co., Ltd., Tokyo, Japan) was continuously administered and titrated to achieve an activated clotting time of 160 to 200 seconds. Nafamostat mesilate was mainly applied for anticoagulation in cases of ECMO combined with continuous renal replacement therapy and was used in a dose of 0.4–1.5 mg/kg/hour to maintain a partial thromboplastin time of 60–80 seconds [29–32]. Patients who had not been on continuous renal replacement therapy were managed with intravenous unfractionated heparin alone. If there was any evidence of ongoing coagulopathy or hemorrhage, intravenous heparin or nafamostat mesilate was ceased. Daily scrubbing and dressing of the insertion site was done with povidone-iodine solution soap. If lung function did not fully recover during VA ECMO despite the restoration of heart function, then this led to upper body hypoxia (PaO_2_ < 50 mm Hg), the so-called harlequin syndrome. In such cases, the ECMO mode was changed to VAV ECMO, and these cases were excluded from the study [30–32].

### 2.7. Weaning from ECMO Support

Successful weaning was defined as weaning from ECMO support, followed by survival for more than 48 hours [33]. All patients received ECMO for at least 48 hours before weaning was attempted. The criteria included SvO_2_ ≥ 70%, hematocrit of 30%–35%, absence of definite bleeding foci, tamponade or left heart distension, left ventricular ejection fraction ≥ 35%, and normal blood lactic acid levels. Furthermore, it was essential to comprehensively identify that the patient's organ function fully recovered and stabilized from underlying disease. ECMO weaning was attempted when the patient on ECMO was hemodynamically stable by the minimal ECMO blood flow (<20% of total flow) with good recovery of myocardial contractility evidenced by echocardiography. The ECMO blood flow was decreased by 500 to 1,000 mL/min for ECMO weaning. Once cardiac pulsatility and contractility improved, ECMO flow was reduced after optimizing inotropic infusion and ventilator setting. Finally, ECMO was withdrawn when sustained stability was noted in the patient's hemodynamic status [34–38].

### 2.8. Statistical Analysis

Statistical analyses were performed using IBM SPSS software (version 21; IBM Corp., Armonk, NY, USA) and using MedCalc for Windows, version 14.8 (MedCalc Software, Ostend, Belgium). Continuous variables were tested for normality using the Kolmogorov-Smirnov test. Continuous variables showing normality were analyzed using Student's *t*-test and expressed as the arithmetic mean ± standard deviation, and continuous variables not showing normality were analyzed using the Mann–Whitney *U* test and expressed as the median (25–75% interquartile range). Categorical variables were displayed as frequency distribution and evaluated with Pearson's chi-square test or Fisher's exact test. Univariate comparisons between the groups for categorical variables were made using the chi-square test and Fisher's exact test as appropriate. Intergroup comparisons of nonnormally distributed continuous variables were made with the Mann–Whitney *U* test. To avoid type 1 error, Bonferroni post hoc correction (*β*-corrected) was applied to data that were initially deemed statistically significant by multiplying the number of variables by the* P* value. Cox proportional hazards model was used to determine independent predictors of successful weaning. Overall survival was calculated according to the Kaplan-Meier method. Independent predictors of overall survival were also determined by using Cox proportional hazards model. Statistical significance was accepted at the 5% level (*P* < .05). To identify independent factors that were associated with patient death, we used univariate and multivariate stepwise logistic regression models. Multiple logistic regression analysis using backwards stepwise regression was performed. Variables with a level of significance defined as *P* < .20 for univariate logistic regression analysis, as well as clinically important variables, were entered as candidate variables in the multivariate models to assess their viability as independent predictors for postoperative ECMO. The results were reported as odds ratios (OR) with 95% confidence intervals (CI) and relevant *P* values. To assess the predictive power of the logistic regression model, a receiver operating characteristic (ROC) curves were used, and we calculated the area under the curve (AUC). Calibration was assessed using the Hosmer-Lemeshow goodness-of-fit test to compare the numbers of observed and predicted deaths in risk groups for the entire range of death probabilities. Discrimination was assessed using the area under the ROC curves. The areas under the ROC curves were compared by a nonparametric approach. This analysis was also utilized to calculate cutoff values, sensitivity, specificity, and overall correctness. Cumulative survival curves as a function of time were generated by the Kaplan-Meier approach and compared between groups using the log-rank test.

## 3. Results

A total of 135 patients who received VA ECMO at a single institution consisted of 94 (69.63%) males and 41 (30.37%) females, with a mean age of 59.44 ± 16.55 years. All the enrolled patients initially presented with cardiac arrest. Suitable indications for VA ECMO according to the ELSO guidelines were determined on the basis of final diagnosis immediately before discharge, but not initial diagnosis. The demographic and clinical characteristics of the study patients according to in-hospital mortality and ECMO weaning are summarized in Tables 1 and 2. Among the 135 patients, 35 (25.93%) survived and were discharged uneventfully, and the remaining 100 (74.07%) did not survive. The mean ages of the survivors and nonsurvivors were 51.91 ± 17.63 and 62.08 ± 15.40 years, respectively. Compared to the nonsurvivors, the survivors had a lower SAPS II (67.77 ± 20.78 versus 90.29 ± 13.30, *P* < .001), a lower SOFA score (12.62 ± 3.49 versus 15.33 ± 2.28, *P* < .001), a lower predicted death rate (71.11 ± 30.50 versus 94.00 ± 9.36, *P* < .001), a higher ipH (7.14 ± 0.22 versus 6.98 ± 0.15, *P* < .001), and a lower lactate level (7.09 ± 4.93 versus 12.11 ± 4.84, *P* < .001). Furthermore, compared to the unsuccessful weaning group, the successful weaning group had a lower SAPS II (74.90 ± 20.81 versus 90.62 ± 13.56, *P* < .001), a lower SOFA score (13.56 ± 3.35 versus 15.26 ± 2.35, *P* = .001), a lower predicted death rate (78.96 ± 27.28 versus 93.95 ± 9.90, *P* < .001), a higher ipH (7.12 ± 0.20 versus 6.98 ± 0.15, *P* < .001), and a lower lactate level (7.37 ± 4.65 versus 12.44 ± 4.86, *P* < .001). CPR was more frequently performed in the unsuccessful weaning group (85.3% [70/82] versus 60.3% [32/53], *P* = .002), and extracorporeal CPR was more frequently performed in the unsuccessful weaning group (74.3% [61/82] versus 49.0% [26/53], *P* = .005). Cardiac arrest more frequently developed in the unsuccessful weaning group (85.3% [70/82] versus 58.57% [31/53], *P* = .001), in-hospital cardiac arrest more frequently developed in the nonsurvivors (91.0% [61/67] versus 8.9% [6/67], *P* < .001), and out-hospital cardiac arrest more frequently occurred in the survivors (61.7% [21/34] versus 38.2% [13/34], *P* = .001). However, there was no significant difference in the application of continuous renal replacement therapy (CRRT), CPR time, pump type, ECMO type, gender, height, body weight, BMI, anticoagulation, admission route, combined operation, or underlying acute renal failure/hypertension/diabetes mellitus between the successful weaning and unsuccessful weaning groups. Furthermore, there was no significant difference in the level of CK-MB, troponin, blood urea nitrogen (BUN), or Cr between the successful and unsuccessful weaning groups (Tables 1 and 2). After univariate logistic regression analysis of initial factors for VA ECMO support, the SAPS II (OR = 1.079, 95% CI 1.047–1.111, *P* < .001), age (OR = 1.304, 95% CI 1.087–1.565, *P* = .004), ipH (OR = 0.314, 95% CI 0.188–0.525, *P* < .001), hospital stay (OR = 0.863, 95% CI 0.819–0.909, *P* < .001), ECPR (OR = 0.173, 95% CI 0.075–0.399, *P* < .001), acute renal failure (OR = 0.283, 95% CI 0.120–0.665, *P* = .004), cardiac arrest (OR = 0.148, 95% CI 0.062–0.352, *P* < .001), and CPR (OR = 0.166, 95% CI 0.0705–0.3941, *P* < .001) were associated with in-hospital VA ECMO mortality. The other variables were not associated with in-hospital VA ECMO mortality in univariate logistic regression analysis. After multivariate logistic regression analysis of initial factors for VA ECMO support, the SAPS II (OR = 1.101, 95% CI 1.005–1.208, *P* = .04), ipH (OR = 0.001, 95% CI 0.000–0.863, *P* = .045), and hospital stay (OR = 0.814, 95% CI 0.734–0.902, *P* < .001) were associated with in-hospital VA ECMO mortality. The other variables, such as age (*P* = .8256), ECPR (*P* = .51), acute renal failure (*P* = .7018), cardiac arrest (*P* = .9957), or CPR (*P* = .73), were not associated with in-hospital VA ECMO mortality in multivariate logistic regression analysis. To evaluate the in-hospital mortality according to age, patients were stratified into 8 groups according to age. The survivors were noted in younger age groups (*P* = .02, chi-square test [*x*^2^ = 17.102]), and the number of survivors decreased with age (*P* = .003, chi-square test for trend [*x*^2^ = 8.688]) (Table 3). Post hoc analysis was performed by using the Student-Newman-Keuls test, and there was a significant difference between the age groups. Post hoc Student-Newman-Keuls test results for all pairwise comparisons are summarized in Table 3.

**Figure 1:**
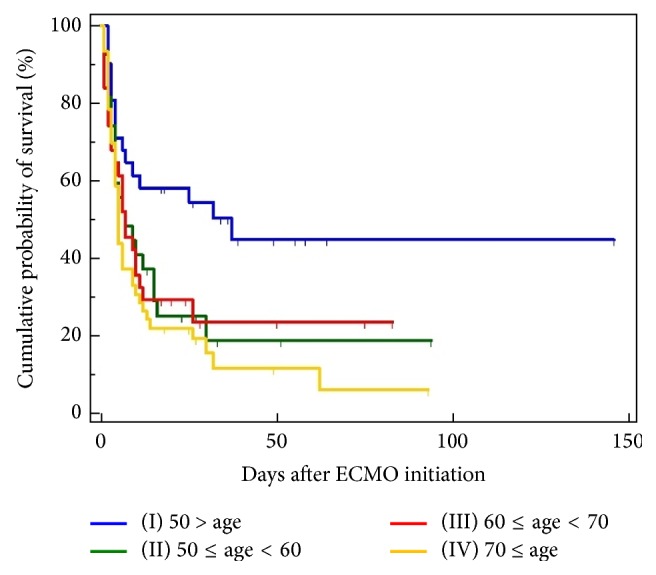
Kaplan-Meier curves for the cumulative survival probability of a pre-ECMO age. To predict in-hospital mortality according to age, patients were divided into 4 groups according to age: group I (age < 50, *n* = 31), group II (50 ≤ age < 60, *n* = 27), group III (60 ≤ age < 70, *n* = 31), and group IV (70 ≤ age, *n* = 46). Comparison of survival curves with the log-rank test: chi-square test *x*^2^ = 11.2779, df = 3, *P* = .0103. Mean, standard error, and 95% confidence intervals for the mean are summarized in Table 2. Comparison of survival probabilities at 6, 10, and 30 days after ECMO initiation with the log-rank test and hazard ratios with 95% confidence intervals is also summarized in Tables 2 and 4. Overall mean survival rate was 25.9%, 6-day survival rate was 50%, 10-day survival rate was 40.7%, and 30-day survival rate was 27.3%.

**Figure 2:**
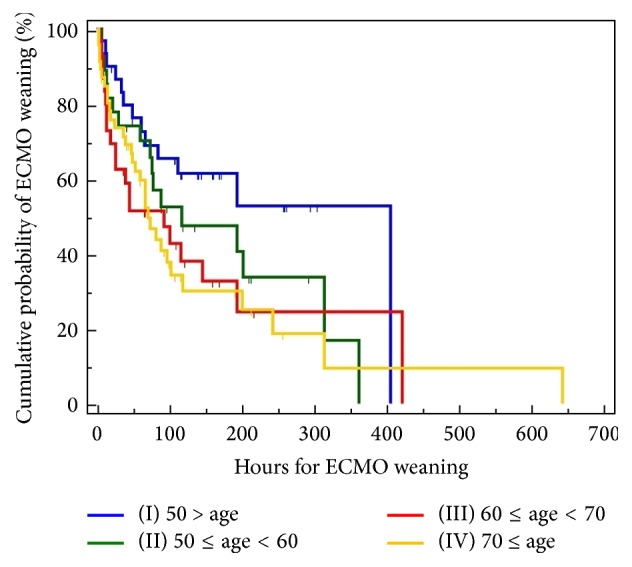
Kaplan-Meier curves for the cumulative ECMO weaning probability of a pre-ECMO age. To predict in-hospital mortality according to age, patients were divided into 4 groups: group I (age < 50, *n* = 31), group II (50 ≤ age < 60, *n* = 27), group III (60 ≤ age < 70, *n* = 31), and group IV (70 ≤ age, *n* = 46). Comparison of survival curves with the log-rank test: chi-square test *x*^2^ = 5.9915, df = 3, *P* = .1120. Mean, standard error, and 95% confidence intervals for the mean are summarized in Table 2. Comparison of ECMO weaning probabilities at 48, 72, and 192 hours after ECMO initiation with the log-rank test and hazard ratios with 95% confidence intervals is also summarized in Tables 2 and 4. Overall mean ECMO weaning rate was 39.20%, 8-day mean ECMO weaning rate was 50%, 10-day mean ECMO weaning rate was 45.8%, and 30-day mean ECMO weaning rate was 37.4%.

To predict in-hospital survival and ECMO weaning, patients were divided into 4 groups according to age: group I (age < 50, *n* = 31), group II (50 ≤ age < 60, *n* = 27), group III (60 ≤ age < 70, *n* = 31), and group IV (age ≥ 70, *n* = 46). The mean, standard error, 95% CI for the mean, median, and 95% CI for the mean about survival are summarized in Table 2. In the Kaplan-Meier analysis, 6-day cumulative survival rates (±standard error) in groups I to IV were 0.67 ± 0.084, 0.51 ± 0.09, 0.51 ± 0.08, 0.37 ± 0.07, and 0.50 ± 0.04, respectively (Figure 1). Furthermore, in the Kaplan-Meier analysis, 30-day cumulative survival rates (±standard error) in group II, group IV, and the entire patients group were 0.18 ± 0.08, 0.15 ± 0.05, and 0.27 ± 0.04, respectively (Table 2). In the Kaplan-Meier analysis, the lower age groups showed higher 6-day, 30-day, and overall cumulative survival rates (comparison of survival curves by the log-rank test: chi-square test *x*^2^ = 11.27, df = 3, *P* = .01). The mean, standard error, and 95% CI for the mean about ECMO weaning are summarized in Table 2. In the Kaplan-Meier analysis, 48-hour cumulative successful ECMO weaning rates (±standard error) in group I, group IV, and the entire patients group were 0.76 ± 0.07, 0.64 ± 0.07, and 0.66 ± 0.04, respectively (Figure 2). Furthermore, in the Kaplan-Meier analysis, 192-hour cumulative survival rates (±standard errors) in group I, group II, group III, and the entire patients group were 0.52 ± 0.11, 0.40 ± 0.10, 0.24 ± 0.10, and 0.36 ± 0.04, respectively (Table 2). In the Kaplan-Meier analysis, the lower age groups showed higher 48-hour, 192-hour, and overall cumulative ECMO weaning rates (comparison of survival curves by the log-rank test: chi-square test *x*^2^ = 5.99, df = 3, *P* = .11). Compared to group I, hazard ratios with 95% CI in groups II to IV were 1.60 with 0.85 to 2.99, 2.04 with 1.07 to 3.89, and 2.05 with 1.16 to 3.63, respectively. Compared to group II, hazard ratios with 95% CI in groups III and IV were 1.27 with 0.65 to 2.50 and 1.28 with 0.70 to 2.34, respectively. Compared to group III, hazard ratios with 95% CI in group IV was 1.00 with 0.54 to 1.86 (Table 4). Mean survival times (with 95% CI for the mean) at the cutoff age of 50 (<50 versus ≥50 years), 60 (<60 versus ≥60 years), 70 (<70 versus ≥70), and 80 (<80 versus ≥80 years) were 48.23 days with 32.72 to 63.74 versus 27.84 days with 16.99 to 38.68, 53.92 days with 36.61 to 71.22 versus 19.30 days with 11.93 to 26.66, 46.70 days with 33.37 to 60.03 versus 16.18 days with 7.64 to 24.72, and 40.05 days with 28.63 to 51.47 versus 6.62 days with 3.80 to 9.44, respectively. The overall survival time was 38.07 days with 27.25 to 48.89. In the Kaplan-Meier analysis, the cutoff ages of 50, 60, and 70 exhibited significant differences in cumulative survival rates (comparison of survival curves by the log-rank test: cutoff 50; chi-square test *x*^2^ = 9.89, *P* = .0017; cutoff 60: chi-square test *x*^2^ = 6.88, *P* = .01; cutoff 70: chi-square test *x*^2^ = 4.58, *P* = .03; cutoff 80: chi-square test *x*^2^ = 2.53, *P* = .11). At the cutoff age of 50, hazard ratio with 95% CI in patients aged ≥50 years was 1.53 with 0.97 to 2.40. At the cutoff age of 60, hazard ratio with 95% CI in patients aged ≥60 years was 1.65 with 1.12 to 2.45. At the cutoff age of 70, hazard ratio with 95% CI in patients aged ≥60 years was 1.53 with 0.97 to 2.40. At the cutoff age of 70, hazard ratio with 95% CI in patients aged ≥60 years was 1.73 with 0.69 to 4.33.

## 4. Discussion

Actually, VA ECMO is an option for circulatory support, and various ventricular assist devices implanted both surgically and percutaneously are other options. The benefits of VA ECMO over other options for circulatory support could be summarized as follows: (1) being an easy method for emergent insertion, (2) potential effectiveness in biventricular support, and (3) capability to simultaneously provide respiratory support [20]. Furthermore, VA ECMO provides adequate temporary perfusion and oxygenation to organs in cardiac arrest patients [21, 23]. VA ECMO might be used as a bridge to myocardial recovery and heart transplantation or a permanent ventricular assist device. Despite all these advantages, the hospital mortality rates of patients who received ECMO support have been reported to be approximately 60% [39–41]. In the recent cohorts with post-acute myocardial infarction (AMI) cardiogenic shock, it has been reported that advanced age, female gender, myocardial infarction, onset-to-percutaneous coronary intervention (PCI) time, evidence of end-organ hypoperfusion, left main coronary disease or total occlusion of the left anterior descending artery, 3-vessel coronary artery disease, hypoxic brain damage, and decreased renal function are independent negative factors for increased in-hospital mortality. However, since these results are limited to patients with post-AMI cardiogenic shock, they could not be generally applied to those with cardiogenic shock on VA ECMO support [42–47]. The selection of appropriate candidates is especially important for successful ECMO treatment, and ECMO treatment requires specialized medical staff and equipment. Thus, outcome prediction for ECMO is mandatory, though it requires enormous expenditure. Outcome prediction for ECMO is also valuable because it is associated with the ethical problem of whom must be cared for with ECMO support. Another important perspective is the emergent nature of ECMO treatment, for which we may have difficulty in comprehensive discussion as to whether ECMO should be initiated or not [48–50].

To make impartial scoring systems for identifying appropriate patient candidates for ECMO, Schmidt et al. [51, 52] evaluated the predicting death for severe acute respiratory distress syndrome (ARDS) on VV ECMO (PRESERVE) and respiratory extracorporeal membrane oxygenation survival prediction (RESP) scores in their survival prediction model for patients who received ECMO support in the intensive care unit (ICU). Klinzing et al. [53, 54] demonstrated that the PRESERVE and RESP scoring systems fail to predict mortality for patients receiving VA ECMO. The EuroSCORE was designed to predict the mortality of patients undergoing cardiac surgery and may correlate with the outcomes of postcardiotomy failure patients. The same study assessed patients treated with ECMO for refractory postcardiotomy shock and found that a EuroSCORE of >20% is associated with mortality [55]. Classically, renal function is an important factor for ECMO survival and intimately related to metabolic acidosis and a high lactate level [56–58]. In our study, factors associated with renal injury, such as Cr or BUN, showed little statistical significance, and increased lactate levels were closely related to prediction of mortality in the study patients, but the SAPS II or ipH was not so [59, 60]. Mehta et al. [61, 62] suggested that blood pressure is a key bedside tool to predict postoperative dialysis risk in patients undergoing cardiac surgery. Damaged cardiac function leads to low cardiac output and then hypoperfusion, subsequently precipitating prerenal acute kidney injury [63, 64].

In a study conducted by Klinzing et al. [53], age of >75 years was an absolute contraindication to ECMO therapy. Furthermore, in the PRESERVE score proposed by Schmidt et al. [52], advanced age is classified as a very high mortality risk factor (ages < 45, 0 point; ages 45–55, 2 points; and ages > 55, 3 points), whereas in the RESP score proposed by Schmidt et al. [51], advanced age is also categorized as a very high mortality risk factor (ages < 50, 0 point; ages 50–59, −2 points; and ages ≥ 60, −3 points). In the score proposed by Roch et al. [65], age of ≥45 is also classified as high in-hospital mortality. As such, most scoring systems concerning ECMO regard advanced age as an absolute contraindication to ECMO or a very high-risk factor and sometimes recommend never to perform ECMO in old age. Recently, Schmidt et al. [47] also reported new scoring system using 12 pre-ECMO parameters, called the survival after VA ECMO (SAVE) score, to identify pre-ECMO factors which influence survival rate in refractory cardiogenic shock patients requiring ECMO. In the SAVE score system proposed by Schmidt et al. [47], advanced age is also classified as a very high mortality parameter (ages 18–38, 7 scores; ages 39–52, 4 scores; ages 53–62, 3 scores; and ages ≥ 63, 0 scores). In 2016, Chen et al. [66] assessed that the SAVE score is a more acceptable scoring system to predict 90-day mortality for patients who received VA ECMO support in the emergency department rather than PRESERVE or RESP score system and an independent variable in the Cox proportional hazards regression model. They also reported that the combination of blood lactic acid level and SAVE score, termed the modified SAVE score, shows more improved discrimination of outcome predictions for patients who receive VA ECMO support in the emergency department [66, 67].

Although many studies reported that advanced age is a great risk factor in VA ECMO mortality, unfortunately there are no definite risk measurement tools that can predict the probability of survival in patients requiring VA ECMO. In our study, patients at younger age showed a higher survival rate with statistical significance (*P* = .02, chi-square test: *x*^2^ = 17.102), and the number of survivors was significantly decreased with age (*P* = .003, chi-square test for trend: *x*^2^ = 8.688). Furthermore, in our study, there were significant differences in survival between age groups, and older age showed a lower survival rate with statistical significance (*P* = .01). By univariate logistic regression analysis, age was significantly associated with in-hospital mortality (OR = 1.30, *P* = .004), whereas, by multivariate logistic regression analysis, it was not significantly associated with in-hospital mortality (*P* = .83). Furthermore, age was not significantly associated with VA ECMO weaning (*P* = .11). Additionally, the multivariate Cox regression analysis showed that age is not a significant predictor of hospital mortality or VA ECMO weaning (*P* = .86 and *P* > .99, resp.). Therefore, it is thought that an age criterion is not a main significant variable for predicting in-hospital mortality or VA ECMO weaning. As previously mentioned, advanced age is not a definite criterion for ECMO treatment, and thus most studies have adopted self-made age criteria, especially advanced age. Thus, further studies on advanced age are needed to confirm our results.

Furthermore, emerging cardiopulmonary assist technique enables physicians to have an opportunity for further evaluation and diagnosis, thereby facilitating appropriate ECMO treatment [68–70]. Therefore, ECMO should be primarily considered in high-risk acute myocardial infarction (AMI) patients with cardiogenic shock or cardiac arrest, regardless of age. In fact, advanced age is the most common risk factor for increased morbidity and mortality in many disease entities and delays or obstructs full recovery from underlying disease [71–77]. However, candidates for VA ECMO should be determined with biological age, but not with calendar age itself. Prompt evaluation and decision are essential to patients who require VA ECMO. It is very difficult to establish criteria for appropriate ECMO and to evaluate whether patients with acute cardiopulmonary failure would respond to conventional treatment. Advanced age is a significant risk factor for VA ECMO treatment; however, a more important factor is physiologic status, but not age itself. Thus, patients of advanced age should not be excluded from the chance of recovery after VA ECMO treatment. Although the great predictive value of scoring systems will remain one of the biggest challenges to physicians, the scoring systems could facilitate communication of objective prognostic information for decision-making by family members and surrogates and may help physicians increase the chance of patient survival and avoid a waste of healthcare services.

Our study has several limitations. First, it was conducted at a single institution, which limited the generalizability of study results. Secondly, our study population was relatively small and had multiple underlying diseases indicated for VA ECMO. Thirdly, despite a study about VA ECMO, a relatively small number of patients with refractory septic shock were enrolled in the study. Fourthly, since our study only focused on VA ECMO, it is difficult to generalize our results to other forms of ECMO, such as VV ECMO. Further studies using various forms of ECMO are warranted. Our study only focused on initial modalities at the decision point of ECMO, and long-term outcomes were not evaluated. evaluated. Fifthly, serum biomarkers, such as brain natriuretic peptide, were not measured in our study. The brain natriuretic peptide is known as a predictor of the outcomes after severe cardiac failure [78]. Further studies are needed to determine whether our results could be accurately applied to such patients. Finally, we performed retrospective analysis, so that additional prospective multicenter studies are needed to confirm our results. Future research should develop more simplified VA ECMO scoring systems with a larger sample size to accurately predict VA ECMO mortality.

## 5. Conclusion

Despite established ELSO indications in ECMO, cardiac indications for VA ECMO may differ greatly among physicians and centers; therefore, it is absolutely important to determine which patients should be treated with VA ECMO. In most reports on ECMO treatment, advanced age is classified as an absolute contraindication to VA ECMO application, so that VA ECMO is not recommended for patients of advanced age. Since ECMO support for adult populations with refractory cardiogenic shock has been exponentially increasing, a comprehensive analysis of risk factors associated with advanced age must be completed. In most studies about VA ECMO, advanced age is regarded as a main significant risk factor and contraindication to VA ECMO treatment; however, a more important factor is physiologic status, but not age. Therefore, patients of advanced age should not be excluded from the chance of recovery with VA ECMO treatment. Additionally, advanced age could be regarded as a major risk factor for VA ECMO; however, it should not be considered an absolute contraindication to VA ECMO. Furthermore, patients who need VA ECMO treatment should be evaluated with impartial predicting systems based on physiologic variables other than age itself.

## Figures and Tables

**Table 1:** Demographic and clinical characteristics of the study patients according to in-hospital mortality and ECMO weaning.

	All patients (*n* = 135)	Survivor or nonsurvivor			Successful or failed weaning from ECMO		
		Survivors	Nonsurvivors	*P*	Successful weaning	Failed weaning	*P*
		(*n* = 35)	(*n* = 100)		(*n* = 53)	(*n* = 82)	
Age (years)	59.44 ± 16.55	51.91 ± 17.63	62.08 ± 15.40	.002	55.43 ± 18.67	62.03 ± 14.57	.02
Gender (M/F)	94/41	27/8	67/35	.36	38/15	56/26	.82
Height (cm)	164.10 ± 8.73	166.68 ± 7.60	163.20 ± 8.95	.04	165.02 ± 7.98	163.51 ± 9.18	.33
Body weight (kg)	66.46 ± 11.07	68.62 ± 11.41	65.70 ± 10.90	.18	67.75 ± 10.88	65.62 ± 11.17	.28
Body mass index (kg/m^2^)	24.61 ± 3.36	24.69 ± 3.93	24.58 ± 3.16	.87	24.85 ± 3.52	24.46 ± 3.26	.51
Body mass index (BMI)				.38			.90
Normal (18.5 ≤ BMI ≤ 22.9)	50 (37.0%)	15	35		18	32	
Overweight (23 ≤ BMI ≤ 24.9)	34 (25.2%)	7	27		14	20	
Obese (25 ≤ BMI ≤ 29.9)	45 (33.3%)	10	35		18	27	
Highly obese (BMI ≥ 30)	6 (4.4%)	3	3		3	3	
ECMO type				.18			.43
VA ECMO	129 (95.6%)	32	97		50	79	
VV-VA ECMO	3 (2.2%)	2	1		2	1	
VA-VV ECMO	1 (0.7%)	0	1		0	1	
VAV ECMO	1 (0.7%)	1	0		1	0	
VA-VAV ECMO	1 (0.7%)	0	1		0	1	
Anticoagulation				.31			.13
With heparin	55 (40.7%)	14	41		23	32	
With nafamostat	74 (54.8%)	21	53		30	44	
No anticoagulation	6 (4.4%)	0	6		0	6	
Primary origin				.27			.56
Cardiac origin	105 (77.8%)	25	80		42	63	
Respiratory origin	1 (0.7%)	1	0		1	0	
Sepsis origin	27 (20.0%)	8	19		9	18	
Trauma origin	2 (1.5%)	1	1		1	1	
Admission route				.49			.36
Via ER	84 (62.2%)	24	60		36	48	
Via ward	51 (37.8%)	11	40		17	34	
Arrest occurrence				<.001			.001
Arrest	101 (74.8%)	16	85		31	70	
No arrest	34 (25.2%)	19	15		22	12	
Arrest place				<.001			.001
Out-hospital arrest	34 (25.2%)	10	24		13	21	
In-hospital arrest	67 (49.6%)	6	61		18	49	
No arrest	34 (25.2%)	19	15		22	12	
CPR				<.001			.002
Yes	102 (75.6%)	17	85		32	70	
No	33 (24.4%)	18	15		21	12	
CPR time (min)	54.69 ± 39.55	34.41 ± 21.15	58.75 ± 41.18	.02	45.50 ± 42.00	58.90 ± 37.95	.11
ECPR				<.001			.005
Yes	87 (64.4%)	12	75		26	61	
No	48 (35.6%)	23	25		27	21	
Operation				.38			.48
No OP	122 (90.4%)	30	92		47	75	
ECMO after OP	4 (3.0%)	2	2		2	2	
OP with ECMO	2 (1.5%)	0	2		0	2	
OP after ECMO	7 (5.2%)	3	4		4	3	
HD (days)	17.0 ± 22.66	43.4 ± 28.25	7.8 ± 9.42	<.001	33.4 ± 27.41	6.4 ± 8.70	<.001
ICU stay (days)	11.4 ± 13.69	24.1 ± 18.59	7.0 ± 7.62	<.001	20.6 ± 16.87	5.4 ± 5.95	<.001
CRRT				.47			.54
Yes	97 (71.9%)	23	74		36	61	
No	38 (28.1%)	12	26		17	21	
IABP				.04			.11
Yes	22 (16.3%)	4	18		6	16	
No	111 (82.2%)	29	82		45	66	
Yes after ECMO weaning	2 (1.5%)	2	0		2	0	
ARF				.005			.35
Yes	64 (47.4%)	9	55		22	42	
No	71 (52.6%)	26	45		31	40	
ECMO time (hours)	99.6 ± 103.23	124.1 ± 76.69	91.0 ± 110.09	.10	128.13 ± 82.20	81.14 ± 111.42	.01
Hypertension				.23			.11
Yes	56 (41.5%)	11	45		17	39	
No	79 (58.5%)	24	55		36	43	
DM				.06			.10
Yes	51 (37.8%)	8	43		15	36	
No	84 (62.2%)	27	57		38	46	
SAPS II	84.45 ± 18.40	67.77 ± 20.78	90.29 ± 13.30	<.001	74.90 ± 20.81	90.62 ± 13.56	<.001
SOFA	14.62 ± 2.89	12.62 ± 3.49	15.33 ± 2.28	<.001	13.56 ± 3.35	15.26 ± 2.35	.001
PDR	88.06 ± 20.05	71.11 ± 30.50	94.00 ± 9.36	<.001	78.96 ± 27.28	93.95 ± 9.90	<.001
iCK-MB	45.30 ± 112.77	24.25 ± 75.01	52.66 ± 122.76	.20	24.94 ± 66.54	58.46 ± 133.23	.09
iTroponin	13.51 ± 47.90	7.21 ± 21.03	15.79 ± 54.36	.37	5.99 ± 17.63	18.56 ± 59.85	.14
iBUN	24.36 ± 20.07	20.96 ± 21.27	25.56 ± 19.61	.24	22.48 ± 19.59	25.58 ± 20.41	.38
iCr	1.90 ± 2.28	1.99 ± 3.64	1.86 ± 1.58	.78	1.94 ± 3.00	1.87 ± 1.68	.86
ipH	7.02 ± 0.19	7.14 ± 0.22	6.98 ± 0.15	<.001	7.12 ± 0.20	6.98 ± 0.15	<.001
Lactate	10.70 ± 5.33	7.09 ± 4.93	12.11 ± 4.84	<.001	7.37 ± 4.65	12.44 ± 4.86	<.001
BNP	712.12 ± 1111.37	525.29 ± 1164.03	798.35 ± 1090.82	.39	816.68 ± 1462.18	646.40 ± 838.05	.58
pCK-MB	191.77 ± 248.01	175.80 ± 256.60	197.36 ± 246.01	.66	212.74 ± 279.29	178.21 ± 226.29	.43
pTroponin	70.49 ± 115.98	50.03 ± 94.51	77.65 ± 122.23	.23	68.32 ± 102.38	71.89 ± 124.57	.86
pBUN	37.60 ± 33.05	34.84 ± 20.46	38.56 ± 36.49	.57	43.59 ± 29.84	33.72 ± 34.60	.09
pCr	2.17 ± 1.81	2.15 ± 2.17	2.17 ± 1.68	.94	2.33 ± 1.97	2.06 ± 1.70	.39
pTB	4.76 ± 7.58	4.61 ± 5.78	4.81 ± 8.14	.90	6.29 ± 7.34	3.77 ± 7.61	.06

BMI, body mass index; ECMO, extracorporeal membrane oxygenation; AMI, acute myocardial infarction; ER, emergency room; CPR, cardiopulmonary resuscitation; E-CPR, extracorporeal cardiopulmonary resuscitation; OP, operation; HD, hospitalization day; ICU, intensive care unit; CRRT, continuous renal replacement therapy; IABP, intra-aortic balloon pump; ARF, acute renal failure; DM, diabetes mellitus; SAPS II, simplified acute physiology score II; SOFA, sepsis-related organ failure assessment; PDR, predicted death rate; BUN, blood urea nitrogen; Cr, creatinine; CK-MB, creatine kinase MB isoenzyme; TB, total bilirubin; BNP, brain natriuretic peptide.

**Table 2:** Mean, standard error, and 95% CI of survival days and ECMO weaning hours according to the age. Cumulative survival rates at 6, 10, and 30 days after ECMO initiation according to the age and cumulative ECMO weaning rates at 48, 72, and 192 hours after ECMO initiation according to the age subgroups.

	Age < 50	50 ≤ age < 60	60 ≤ Age < 70	Age ≥ 70	Overall
Mean survival days	48.2	22.0	26.7	15.9	38.1
SE of survival days	7.9	6.1	7.0	4.0	5.5
95% CI of survival days	7.00–37.00	4.00–15.00	4.00–11.00	4.00–9.00	5.00–10.00

Cumulative survival rates in 6 days					
SP	0.677	0.519	0.516	0.370	0.504
SE	0.084	0.096	0.089	0.071	0.043
Cumulative survival rates in 10 days					
SP	—	0.407	0.355	0.304	0.407
SE	—	0.094	0.085	0.067	0.042
Cumulative survival rates in 30 days					
SP	—	0.18	—	0.152	0.273
SE	—	0.083	—	0.058	0.040

Mean ECMO weaning hours	224.6	165.8	149.3	126.6	175.6
SE of ECMO weaning hours	30.0	28.6	34.3	22.7	21.7
95% CI of ECMO weaning hours	165.30–283.01	109.81–221.78	81.99–216.58	82.07–171.04	133.04–218.17

Cumulative ECMO weaning rates in 48 hours					
SP	0.762	—	—	0.644	0.662
SE	0.078	—	—	0.071	0.041
Cumulative ECMO weaning rates in 72 hours					
SP	—	—	—	0.468	0.569
SE	—	—	—	0.077	0.044
Cumulative ECMO weaning rates in 192 hours					
SP	0.527	0.406	0.245	—	0.366
SE	0.113	0.109	0.101	—	0.049

ECMO, extracorporeal membrane oxygenation; CI, confidence interval; SE, standard error; SP, survival proportion.

**Table 3:** In-hospital mortality according to age.

	All patients	Survivors	Nonsurvivors	*P*
	(*n* = 135)	(*n* = 35)	(*n* = 100)	.0167
Age < 50	31 (23.0%)	15	15	
50 ≤ age < 55	17 (12.6%)	2	15	
55 ≤ age < 60	10 (7.4%)	4	6	
60 ≤ age < 65	12 (8.9%)	2	10	
65 ≤ age < 70	19 (14.1%)	6	13	
70 ≤ age < 75	27 (20.0%)	5	22	
75 ≤ age < 80	9 (6.7%)	1	8	
Age ≥ 80	10 (7.4%)	0	10	

*P*, chi-square test: *x*^2^ = 17.102, *P* = .0167, chi-square test for trend: *x*^2^ = 8.688, *P* = .0032.

**Table 4:** Hazard ratios with 95% confidence intervals about survival probability and ECMO weaning probability according to age.

	Age < 50	50 ≤ age < 60	60 ≤ age < 70	Age ≥ 70
	Hazard ratios with 95% confidence intervals about survival probability according to age			
Age < 50	—	1.87 0.99 to 3.54	2.90 1.70 to 4.97	5.71 3.38 to 9.65
50 ≤ age < 60	0.53 0.28 to 1.01	—	1.55 0.83 to 2.90	3.05 1.65 to 5.63
60 ≤ age < 70	0.34 0.20 to 0.59	0.65 0.35 to 1.21	—	1.97 1.18 to 3.27
Age ≥ 70	0.18 0.10 to 0.30	0.33 0.18 to 0.61	0.51 0.31 to 0.84	—

	Hazard ratios with 95% confidence intervals about ECMO weaning probability according to age			
Age < 50	—	1.60 0.86 to 3.00	2.0 1.08 to 3.89	2.06 1.16 to 3.63
50 ≤ age < 60	0.62 0.33 to 1.17	—	1.28 0.65 to 2.50	1.28 0.70 to 2.34
60 ≤ age < 70	0.49 0.2570 to 0.9268	0.78 0.40 to 1.53	—	1.00 0.54 to 1.86
Age ≥ 70	0.4865 0.28 to 0.86	0.78 0.43 to 1.42	1.00 0.54 to 1.85	—

ECMO, extracorporeal membrane oxygenation.

## Abbreviations

ECMO: Extracorporeal membrane oxygenation
VA: Venoarterial
ELSO: Extracorporeal life support organization
ECLS: Extracorporeal life support
IABP: Intra-aortic balloon pump
CPB: Cardiopulmonary bypass
VV: Venovenous
CESAR: Conventional ventilatory support versus extracorporeal membrane oxygenation for severe adult respiratory failure
VAV: Venoarterial venous
BMI: Body mass index
ECPR: Extracorporeal cardiopulmonary resuscitation
CPR: Cardiopulmonary resuscitation
ICU: Intensive care unit
EM: Emergency department
SOFA: Sepsis-related organ failure assessment
SAPS II: Simplified acute physiology score II
AKIN: Acute kidney injury network
RIFLE: Risk, injury, failure, loss of kidney function, and end-stage kidney disease
Cr: Serum creatinine
PMP: Polymethylpentene
ARDS: Acute respiratory distress syndrome
PRESERVE: Predicting death for severe acute respiratory distress syndrome on VV ECMO
RESP: Respiratory extracorporeal membrane oxygenation survival prediction
SAVE: Survival after VA ECMO
AMI: Acute myocardial infarction
OR: Odds ratios
CI: Confidence intervals
ROC: Receiver operating characteristic
AUC: Area under the curve.
